
JOURNAL INFORMATION
==============================
NLM Title Abbreviation: ScientificWorldJournal
Journal ID: ScientificWorldJournal
NLM Journal ID: 101131163
Journal ID: TSWJ

The Scientific World Journal

ISSN: 2356-6140
EISSN: 1537-744X
Publisher: Wiley

ARTICLE INFORMATION
==============================
PMCID: PMC6009368
PMID: 29973844
DOI: 10.1100/tsw.2002.67
Article ID: 384101
Article version: 1
Subjects: Short Report

Structural Insight into the Mechanisms of Targeting and Signaling of Focal Adhesion Kinas

Liu Gaohua 1
Guibao Cristina D. 1
Zheng Jie 2 *
Department of Structural Biology MS 311 St. Jude Children's Res. Hospital, Memphis, TN 38103, USA
Department of Molecular Sciences, University of Tennessee Health Science Center, Memphis, TN 38163, USA
*Jie Zheng: jie.zhang@stjude.org
Publication date: 2002
Electronic publication date: 2002 Apr 22
Volume: 2
First page: 142
Last page: 143
Copyright: Copyright © 2002 Gaohua Liu et al.
Copyright year: 2002
License: This is an open access article distributed under the Creative Commons Attribution License, which permits unrestricted use, distribution, and reproduction in any medium, provided the original work is properly cited.
License URL: https://creativecommons.org/licenses/by/3.0/

Funding: American Heart Association 0051073B

==============================
Advances in Gene Technology: The Genome and Beyond –
Structural Biology for Medicine (Proceedings of the 2002 Miami
*Nature Biotechnology* Winter Symposium)
*TheScientificWorld* 2002, 2(S2), 142–143
ISSN 1532-2246; DOI 10.1100/tsw.2002.67

### STRUCTURAL INSIGHT INTO THE MECHANISMS OF TARGETING AND SIGNALING OF FOCAL ADHESION KINASE

Gaohua Liu[†], Cristina D. Guibao[†], and Jie Zheng[†,‡,*]
[†]Department of Structural Biology, St. Jude Children' Research Hospital Memphis, TN 38105;
[‡]Department of Molecular Sciences, University of Tennessee Health Science Center, Memphis, TN 38163
*Jie.Zhang@stjude.org

**INTRODUCTION.** Focal adhesion kinase (FAK) is a nonreceptor tyrosine kinase whose focal adhesion targeting (FAT) domain interacts with other focal adhesion molecules in integrin-mediated signaling. Localization of activated FAK to focal adhesions is indispensable for its function. To help understand the mechanisms of focal adhesion targeting of the FAT domain, we have elucidated the solution structure of the FAT domain of FAK in the context of the complex formed by the FAT domain bound to a peptide corresponding to the LD2 sequence (residues 139 to 162) of chicken paxillin, a FAK-binding partner.

**METHOD**. The cDNA encoding the FAT domain (residues 916-1053) was subcloned into a PET28a vector. The N-terminal His-tagged FAT domains were subsequently expressed in *E. coli*. Sample concentrations for NMR experiments were typically 0.5 to 1.6 mM in 10 mM potassium phosphate buffer (pH 6.5) and 0.1% $NaN_3$. The molar ratio between the FAT domain and the LD2 peptide) in the NMR samples was 1:10. All NMR data were acquired with Varian Inova 600-Mhz spectrometers at 37 °C. The program DYANA[1] was used to calculate the structures. The 25 structures with the lowest target functions were included in the final families.

**RESULTS.** The FAT domain has four α-helices and comprises an elongated “right-turn” helical bundle (Fig. 1). In antiparallel helix packing, all four helices (H1 to H4) are connected by short “underhand” loops. Apart from helix H4, all helices are relatively straight. The lengths of the four helices differ, H4 is the longest and contains a kink in the middle (around Leu1035). Almost all the residues sequestered inside the bundle are hydrophobic, and the helix bundle is therefore mainly stabilized by hydrophobic core. We observed extensive interhelical interactions among these hydrophobic residues, not only between neighbor helices, but also between cross-diagonal helices (H1-H3 and H2-H4). However, our examination of the side chains of charged residues revealed that few inter-helical salt bridges are present. Each of the three "underhand" loops that connect the four helices in the FAT domain is short and highly structured.

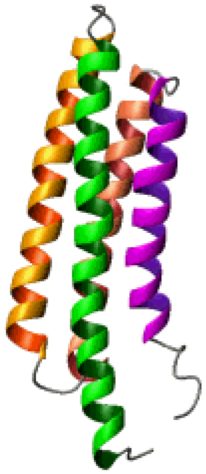

**FIGURE 1**. Ribbon diagram of the structure of the FAT domain.

**DISCUSSION.** We found that the structure of the FAT domain resembles that of the C-terminal tail domain of the focal adhesion protein vinculin[2]. The structures of helices H1 to H4 of the FAT domain are similar to the C-terminal four helices (H2, H3, H4, and H5) of the tail domain of vinculin. The homology to vinculin is particularly interesting; vinculin is also colocalized in the focal adhesions, and its tail domain also binds to paxillin. NMR analyses of the structure of the FAT domain revealed several features that are likely to be important in its function. One intriguing feature of the FAT domain is that hydrophobic interaction is the chief force that holds the four helices together. Few salt bridges between the helices are present. Therefore, interrupting the core hydrophobic interactions by replacing hydrophobic residues with hydrophilic residues will probably disrupt the 3D structure–completely. Indeed, the structure of dominant-negative mutant of chicken FAK is substantially altered by the mutation Leu1035Ser (Leu1034 in human and mouse sequence)[3], and this dominant-negative protein cannot localize to focal adhesions. We hypothesize that the hydrophobic core will collapse and that the structure of the FAT domain will be completely altered if a hydrophilic residue (e.g., serine) replaces Leu1035. Because the four-helix bundle of the FAT domain is mainly maintained by hydrophobic interactions, the FAT domain may exist in an "open" form under certain physiologic conditions.

**ACKNOWLEDGMENT.** We thank Dr. J. Thomas Parsons for kindly providing the chicken FRNK cDNA. This work was supported by the American Lebanese Syrian Associated Charities and a grant-in-aid from the American Heart Association (0051073B).

**REFERENCES**

1. Guntert, P., Mumenthaler, C., and Wuthrich, K. (1997) J. Mol. Biol. 273, 283–298.
2. Bakolitsa, C., de Pereda, J.M., Bagshaw, C.R., Critchley, D.R., and Liddington, R.C. (1999) Cell 99, 603–613.
3. Hauck, C.R., Klingbeil, C.K., and Schlaepfer, D.D. (2000. Immunol. Res. 21, 293–303.
